# Sex differences in mitochondrial function in aging mouse skeletal muscle

**DOI:** 10.3389/fragi.2026.1824237

**Published:** 2026-04-29

**Authors:** Angelina Holcom, Ashley Liao, Kaitlyn G. Holden, Anne M. Bronikowski, Ashley E. Webb

**Affiliations:** 1 Buck Institute for Research on Aging, Novato, CA, United States; 2 IISAGE Consortium, Novato, CA, United States; 3 Department of Integrative Biology, W. K. Kellogg Biological Station, Michigan State University, Hickory Corners, MI, United States

**Keywords:** alkaline comet assay, flexor digitorum brevis (FDB), mitochondria bioenergetics, mitochondrial DNA copy number, sex differences, skeletal muscle aging

## Abstract

**Introduction:**

Sex differences in lifespan and age-associated phenotypes are pervasive across species, yet the mechanisms remain poorly understood. Mitochondrial dysfunction is a major hallmark of aging, but whether skeletal muscle mitochondria age along sex specific trajectories remains incompletely defined.

**Methods:**

Here, we profiled mitochondrial bioenergetics and DNA integrity in flexor digitorum brevis (FDB) muscle from young (3–4 months) and aged (20–24 months) male and female C57BL/6 mice. We quantified cellular respiration in intact myofibers, measured mitochondrial DNA (mtDNA) copy number, and assessed expression of genes involved in mitochondrial dynamics, electron transport chain (ETC) function, and mtDNA maintenance.

**Results:**

Cellular respiration differed by sex at baseline and changed with age in a sex-dependent manner. Aged females exhibited a lower basal and ATP-linked respiration than aged males. In contrast, spare respiratory capacity increased in aged females relative to aged males, consistent with age- and sex-specific remodeling of the bioenergetic reserve. mtDNA copy number increased with age in both sexes, with a greater increase in mtDNA content in aged males. Gene-expression analyses revealed age- and/or sex-dependent changes, including lower *Pink1* expression in females compared to males, an age-related increase in the mtDNA maintenance gene *Polg2* only in males, though most genes were not significantly different. As an exploratory systemic readout, we additionally assessed DNA damage responsiveness in whole-blood leukocytes using the alkaline comet assay following oxidative challenge; young females exhibited greater induced DNA damage than young males.

**Discussion:**

Together, these data define sex- and age-associated mitochondrial remodeling in FDB and provide an initial assessment of sex-dependent inducible DNA damage responses in blood, underscoring the importance of sex as a biological variable in studies of aging.

## 1 Introduction

Aging is frequently accompanied by a progressive decline in skeletal muscle mass and function that increases frailty risk and contributes to impaired mobility and loss of independence (Cruz-Jentoft et al., 2019; Larsson et al., 2019). Termed sarcopenia, this phenotype reflects both reduced muscle quantity and quality, and skeletal muscle is among the earliest tissues to exhibit measurable age-associated functional changes (Distefano and Goodpaster, 2018).

A central hallmark of skeletal muscle aging is mitochondrial dysfunction, including age-related alterations in oxidative phosphorylation capacity, mitochondrial biogenesis, mitochondrial DNA (mtDNA) integrity, and the regulatory pathways that govern mitochondrial dynamics and quality control (e.g., fusion/fission and mitophagy) (Leduc-Gaudet et al., 2021; Peterson et al., 2012; Springer-Sapp et al., 2025). In parallel, aging is associated with increased nuclear and mitochondrial DNA lesions and impaired genome maintenance across tissues and cell types, which can disrupt transcriptional output and proteostasis and ultimately compromise performance (Bou Saada et al., 2017).

Despite substantial progress, much of the mitochondrial aging literature in rodents has focused on a limited set of large locomotor muscles (Leduc-Gaudet et al., 2021; Moschinger et al., 2019; Sarver et al., 2024; Springer-Sapp et al., 2025; Triolo et al., 2022). This emphasis may obscure muscle-specific trajectories, particularly because skeletal muscle does not age uniformly across anatomical locations and fiber-type compositions (Chai et al., 2011; Naruse et al., 2023; Titova et al., 2025). Across mammals, sex differences in lifespan and aging trajectories are common, but they are not uniform: females outlive males in humans and many primates, whereas in some species males are longer-lived, and sex differences can be condition-dependent (Austad, 2019; Austad and Fischer, 2016). Here, we used young and aged male and female C57BL/6 mice to test how sex and age shape mitochondrial function in the flexor digitorum brevis.

The flexor digitorum brevis (FDB) is a small foot muscle that is highly tractable for *ex vivo* assays in mature myofibers and provides a complementary window into muscle aging biology beyond commonly studied hindlimb muscles. Work in male mice indicates that FDB myofibers exhibit age-associated remodeling of mitochondrial architecture and function, supporting the use of this muscle as a sensitive model for mitochondrial aging (del Campo et al., 2018). Moreover, denervation and neuromuscular junction remodeling can differ across muscles, which highlights the importance of examining multiple muscle groups when defining mechanisms of muscle aging (Chai et al., 2011). However, the extent to which mitochondrial aging phenotypes in discrete skeletal muscles differ by sex remains incompletely defined.

Here, we examine how age and sex interact to shape cellular respiration, mtDNA copy number, and expression of genes involved in mitochondrial dynamics, electron transport chain function, and mtDNA maintenance in the mouse FDB. As a secondary analysis, we assessed DNA integrity responses to oxidative challenge in whole-blood leukocytes using the alkaline single cell gel electrophoresis (“comet”) assay, providing an accessible systemic readout that complements the primary muscle-focused dataset (Møller et al., 2020). Given reported sex differences in longevity and healthspan measures in C57BL/6 mice, we hypothesized that aging trajectories in bioenergetic capacity and inducible DNA damage responses would differ between males and females (Baumann et al., 2019). Together, these data provide a cross-modal assessment of sex- and age-associated differences in muscle bioenergetics and DNA integrity.

## 2 Materials and methods

### 2.1 Animals

All procedures were performed in accordance with institutional animal care guidelines. C57BL/6 mice were obtained from the NIA and Charles River at the indicated ages: young mice were 3–4 months old and aged mice were 20–24 months old at the time of receipt. The average life-span for males is around 878 days and 794 days for females (Kunstyr and Leuenberger, 1975). Upon arrival, mice were acclimated for 7 days in the Buck Institute’s vivarium prior to experimentation.

Estrous-cycle stage was not assessed or synchronized for female mice. Females were sampled without regard to cycle stage; thus, estrous status was effectively randomized across individuals. Because our primary comparisons were age-associated and sex-associated effects at the group level, and because cycle stage would be expected to introduce within-group variability rather than a consistent directional bias, we did not control for estrous stage in this study.

Mice were housed under controlled temperature on a 12-h light/12-h dark cycle with *ad libitum* access to standard laboratory chow and water. Mice were euthanized without fasting during the light phase (morning/early afternoon) by CO_2_ according to approved IACUC guidelines.

### 2.2 FDB muscle dissection and myofiber isolation

FDB muscles were dissected from both hind limbs using established methods (Schuh et al., 2012; Tarpey et al., 2018).

Isolation of single fibers was performed according to established methods (Agilent Technologies, Inc, 2016; Schuh et al., 2012; Tarpey et al., 2018). Muscles were placed in a well plate with warmed dissociation medium and incubated at 37 °C for 2 hours with 5% O_2_ (Agilent Technologies, Inc., 2016). The tissue was then transferred to a well plate with warmed incubation medium and dissociated manually into single fibers by pipetting 5–10 times (with P1000) (Agilent Technologies, Inc., 2016). Fibers were inspected under a dissection microscope to confirm integrity and minimize breakage.

### 2.3 Mitochondrial DNA content

After FDB muscle fiber isolation, the liquid was removed and tissue was lysed with Qiagen DNeasy Blood and Tissue kit lysis buffer and proteinase K for 1 hour at 56 °C, then after step 2 in the kit’s protocol a 20-gauge needle with syringe was used to further breakdown the tissue. The rest of the kit’s protocol is followed to isolate the DNA. The eluted DNA was then quantified with a nanodrop and diluted to 10 ng/μL with a final volume of 50 µL qPCR was then run with the Power Up SYBR Green kit with in a 96 well qPCR plate. Mitochondrial genes (16S rRNA and ND1) were compared to the nuclear gene HK2 (Quiros et al., 2017). The mitochondrial genome primer (mMito) that amplifies a unique region of the mitochondrial genome was compared to the nuclear gene B2M (Malik et al., 2016). Two complementary analyses were performed: (i) Fold change with ΔΔCt = aged–young (average of male + female Cts) and ΔCt = (mtDNA Ct–nDNA Ct) and (ii) calculating mtDNA copy number using the equation: 2 × 2 ^ (Ct (nDNA) − Ct (mtDNA)). Table 1 includes the list of primers used. For the mtDNA assay the mice were 3 months for young age group and 24 months for the aged group.

**TABLE 1:** Primers for mtDNA qPCR.

Gene	Source	Forward primer 5′-3′	Reverse primer 5′-3′
16s rRNA	Quiros et al. (2017)	CCG​CAA​GGG​AAA​GAT​GAA​AGA​C	TCG​TTT​GGT​TTC​GGG​GTT​TC
ND1	Quiros et al. (2017)	CTA​GCA​GAA​ACA​AAC​CGG​GC	CCG​GCT​GCG​TAT​TCT​ACG​TT
HK2	Quiros et al. (2017)	GCC​AGC​CTC​TCC​TGA​TTT​TAG​TGT	GGG​AAC​ACA​AAA​GAC​CTC​TTC​TGG
mMito	Malik et al. (2016)	CTA​GAA​ACC​CCG​AAA​CCA​AA	CCA​GCT​ATC​ACC​AAG​CTC​GT
B2M	Malik et al. (2016)	ATG​GGA​AGC​CGA​ACA​TAC​TG	CAG​TCT​CAG​TGG​GGG​TGA​AT

### 2.4 Cellular respiration with FDB muscle fibers

Oxygen consumption rate (OCR) was measured in intact single fibers using a Seahorse XFe96 analyzer following Agilent guidelines and published protocols (Agilent Technologies, Inc., 2016; Schuh et al., 2012). For the assay we used 3 month old mice for the young cohort and 24 months for the aged cohort.

After initiation of muscle dissociation, the 96 well seahorse plate was coated with 3–5 µL of a 1:1 mix of DMEM (same used in the dissociation medium) and extracellular matrix (ECM) and allowed to air dry for 10–30 min. After single fiber isolation in incubation medium, 90 µL of the fiber mix was transferred to the seahorse plate (eight technical replicates per mouse) and 60% confluency was confirmed with a dissection microscope. The fibers were left to adhere for 5 minutes. While the fibers were adhering, the seahorse cartridge was prepared with 20 µL of 10 µM oligomycin, 4 µM FCCP, 100 mM sodium pyruvate, and 5μM Rotenone with Antimycin A. The 96 well plate containing the fibers underwent media change for prewarmed assay medium (aCSF) (Agilent Technologies, Inc., 2016) by aspirating and washing with aCSF with a final volume of 160 µL. 160µL of aCSF was also added to the wells without fibers and used for background normalization and sensor calibration. The plate was then incubated at 37 °C without CO_2_ for 1 hour, before running as previously described (Agilent Technologies, Inc., 2016; Schuh et al., 2012).

After the assay completion, protein content was quantified using the Pierce BCA protein assay kit and used to normalize OCR values, as described previously (Schuh et al., 2012). Seahorse assays were performed in three independent batches. Mean protein concentrations ranged from 267–470 μg/mL). For analysis, OCR values from eight technical replicate wells per mouse were averaged to generate a single per-mouse value prior to statistical testing.

### 2.5 RT-qPCR with FDB muscle fibers

After isolating the FDB muscle fibers the liquid was removed and the tissue was lysed with Qiagen RNeasy kit (beta-ME is added to lysis buffer). The tissue and lysis buffer was homogenized for two cycles of 30 s on, 30 s off, then pipetted 15 times with a 20-gauge needle. Then the RNA was eluted following the kit instructions. 50ng RNA was used to produce cDNA using the high-capacity cDNA reverse transcription kit from Applied Biosystems. 0.5 µL of cDNA was used per well with 14.5 µL of master mix for qPCR. Primers were selected from the literature (Baig et al., 2023; He et al., 2021; Reddy et al., 2018). Table 2 includes the list of primers used. The mice used for this assay were 3–4 months old for the young group and the aged group ranged from 20, 21, and 24 months old.

**TABLE 2:** Primers for RT-qPCR.

Gene	Source	Forward primer 5′-3′	Reverse primer 5′-3′
*Drp1/Dnm1l*	NCBI Primer-BLAST	ACC​CGG​AGA​CCT​CTC​ATT​CT	CCA​TTC​TTC​TGC​TTC​AAC​TCC​ATT
*Mfn1*	NCBI Primer-BLAST	GGC​CCT​GTC​CAG​GTG​CAT​AA	TGG​CCG​AAG​ATT​GCA​GTG​ATG
*Tfam*	NCBI Primer-BLAST	GCG​TGC​TAA​AAG​CAC​TGG​G	ACT​TCG​GAA​TAC​AGA​CAA​GAC​TGA
*Beta Actin*	NCBI Primer-BLAST	ACTGTCGAGTCGCGTCCA	ATC​CAT​GGC​GAA​CTG​GTG​G
*Pink1*	NCBI Primer-BLAST	AGT​GGG​ACT​CAG​ATG​GCT​GT	ACT​GGA​GCT​GTT​GAA​AGG​CA
*Atg5*	NCBI Primer-BLAST	TGG​ATG​GGA​CTG​CAG​AAT​GA	GCT​CCG​TCG​TGG​TCT​GAT​A
*ND1*	El-Merhie et al. (2017)	GCT​TTA​CGA​GCC​GTA​GCC​CA	GGG​TCA​GGC​TGG​CAG​AAG​TAA
*Ndufb8*	NCBI Primer-BLAST	GCC​TGC​ATT​CCT​GTT​TTC​CTG	CCC​ATC​CCC​AGT​GAG​ATG​AC
*CO1*	El-Merhie et al. (2017)	TCA​ACA​TGA​AAC​CCC​CAG​CCA	GCG​GCT​AGC​ACT​GGT​AGT​GA
*Parkin*	NCBI Primer-BLAST	CCC​TTC​AGT​CCT​ACC​AAC​CC	TGC​GCA​TAA​ACC​TAT​GGG​GG
*Ppargc1a*	NCBI Primer-BLAST	GCA​TGA​GTG​TGT​GCT​GTG​TG	ACA​TGT​CCC​AAG​CCA​TCC​AG
*Nrf1*	NCBI Primer-BLAST	AAC​TGT​GAA​GCT​GTC​CAG​GG	TGC​TGC​TGG​GAT​CTT​GCT​TT
*Tfb2m*	IDT primer design	GGC​TGA​CCC​TTC​TGT​GTA​AAT​A	GAC​AGG​GTT​TCT​CTG​TGT​ATC​C
*Twnk*	NCBI Primer-BLAST	GGA​TGC​GGA​TCA​CAT​CCA​AG	TCC​CAG​TTA​ATG​GGG​CTG​TG
*Polg2*	El-Merhie et al. (2017)	CTG​GTT​GCG​TCA​TCG​GCT​TC	TGC​TTC​CCT​TGC​GTC​CCA​AT
*Ssbp1*	NCBI Primer-BLAST	GGC​ACG​AAG​TTG​TGT​TTC​CC	GAC​ACT​CGG​ACT​CCA​GCG​AG

### 2.6 Comet assay on whole blood

This assay utilized young mice at 3 months old and aged mice at 20 months old. Cardiac blood was collected with a 25-gauge needle syringe and stored on ice in Vacutainer plastic tubes with K2EDTA (BD). Tubes were inverted 8–10 times after addition of the blood, followed by 100x dilution with PBS. Whole-blood nucleated cells were counted by hemocytometer, and 300,000 cells per animal were embedded in agarose for comet analysis. Because the number of scorable comets per slide was lower than anticipated (i.e., many fields contained few analyzable nucleoids), we used a higher input of cells to increase the likelihood of obtaining sufficient scorable cells for quantification. The number of comets scored per animal ranged from 4 to 41, depending on group and condition (young males: 17–38; young females: 12–24; young males + damage: 4–15; young females + damage: 9–15; aged males: 23–29; aged females: 21–41; aged males + damage: 7–12; aged females + damage: 7–10).

The comet slides were pre-warmed in a 37 °C incubator, and the 0.5% standard agarose and 0.5% low melting point agarose (LMA) were warmed in a heat block at 100 °C for 5 min then reduced to 37 °C for 20 min. Slides were coated with a thin layer of normal agarose and solidified at room temperature.

Cells were mixed into 50 µL of 0.5% LMA and transferred to slides. Damage was induced for 20 min with 100 µM H_2_O_2_ at 4 °C or on ice). Cells were then mixed with 500 µL of 0.5% LMA and transferred to the slide and kept in the dark at 4 °C to solidify.

Slides were placed in cold comet lysis buffer (Table 3) for 60 min at 4 °C in the dark (all of the following procedures were performed in the dark) then moved to cold alkaline unwinding solution for 20 min at 4 °C. Slides were then moved to an electrophoresis box filled with Alkaline unwinding buffer and run at 1 V/cm (based on the distance between electrodes on the box) for 20 min at 4 °C (constant current at 300 mA), then slides were moved to cold neutralizing buffer for 15 min at room temp, then transferred to cold DNA stain for 15 min and rinsed gently with water. Excess liquid was removed before imaging at ×10 magnification with a Nikon SRRF and TIRF fluorescent microscope.

**TABLE 3:** Comet assay solutions (Olive and Banáth, 2006).

Solutions (store at 4 °C)	Ingredients final concentration
Wash Buffer (WB)	100 mL PBS, 1 mM EDTA, 0.1% BSA
Comet Lysis Buffer (pH 10)	200 mL water, 2.5M NaCl, 100 mM EDTA, 10 mM Tris-HCl, 1% Triton X-100, 10% DMSO
Alkaline Unwinding/Electrophoresis Buffer (pH > 13)	300 mM NaOH, 1 mM EDTA
Neutralization Buffer (pH 7.5)	0.4M Tris-HCl
DNA stain	1:10,000 SYBR Gold in TE Buffer (pH 7.5)

### 2.7 Statistics

Statistical analyses were performed in GraphPad Prism (v11). For all datasets, normality was assessed using the Shapiro–Wilk test. Unless otherwise noted, biological replicates (n) refer to individual mice, and technical replicates were averaged to generate a single value per mouse prior to inferential testing. Group comparisons were primarily performed using two-way ANOVA (age × sex) with Tukey’s multiple-comparisons test. For Seahorse measurements, OCR parameters were computed per well (8 technical replicate wells) and then averaged across wells for each mouse and fold-change comparisons were evaluated using unpaired two-tailed t-tests.

For RT–qPCR, technical replicate wells were first quality-controlled based on amplification and melt-curve profiles. Wells with absent/aberrant amplification or melt curves inconsistent with a single specific product were excluded. When one technical replicate failed QC, the remaining replicate was used if it passed QC; samples with no passing technical replicates for a given target were excluded for that target. Ct values from remaining technical replicates were averaged per mouse, and ΔCt and ΔΔCt values were computed using per-mouse averages. After QC-based exclusions, biological outliers were identified on per-mouse values using the ROUT method (Q = 5%) and removed prior to inferential testing. ROUT-based exclusions were as follows: Ssbp1 (1 young male), Polg2 (1 young male), Ndufb8 (1 aged male and 1 aged female), Twnk (1 aged male and 2 aged females), Pink1 (2 young females), and Drp1 (1 young female). Statistical significance was determined after these exclusions.

For secondary analyses of RT–qPCR results collapsed across sex to assess the main effect of age, unpaired two-tailed t-tests were used; when normality assumptions were not met (e.g., pooled Polg2), the Mann–Whitney U test was applied. Potential biological outliers were identified using the ROUT method (Q = 5%) on per-mouse summary values and removed prior to analysis.

For comet assay endpoints, the unit of analysis was the per-mouse summary value (mean) computed from scorable comets. Individual comets with extreme values consistent with technical artifact were excluded prior to calculating per-mouse summaries; excluded comets did not affect biological n.

## 3 Results

### 3.1 Mitochondrial DNA content increases with age in FDB muscle in males and females

**FIGURE 1:**
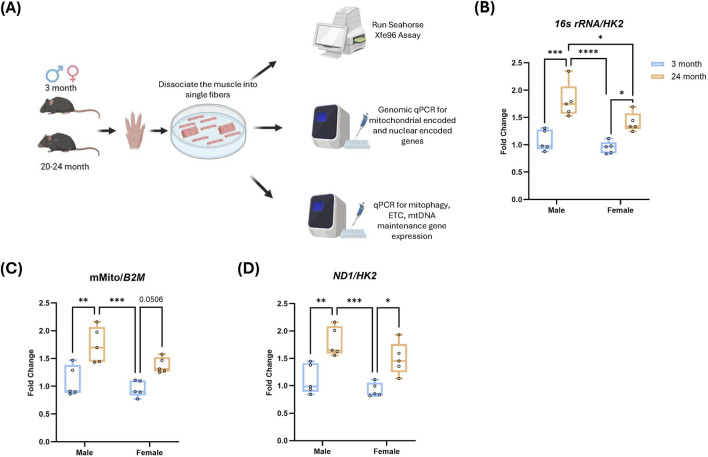
Mitochondrial DNA content increases with age in male and female FDB muscle **(A)** Experimental workflow. Flexor digitorum brevis (FDB) muscles were dissociated into single fibers for Seahorse XFe96 respirometry, mitochondrial DNA qPCR analysis, and gene expression quantification. **(B–D)** Fold change analysis for *16s rRNA*, mMito and *ND1* aged (24-month) male and female mice (normalized to either *HK2* or *B2M*) across groups relative to mean of young mice (3-month). Box plots show median and interquartile range with individual mice overlaid (n = 5 per group). Statistics: two-way ANOVA with Tukey’s multiple comparisons test. *P < 0.05; **P < 0.01; ***P < 0.001; ****P < 0.0001.

We characterized the sex- and age-related differences in FDB muscle from young adult and aged male and female mice. We isolated FDB muscle from both hind limbs and measured differences in mitochondrial DNA (mtDNA) content, respiratory rates, and gene expression (Figure 1A). We started with measurement of changes in mtDNA content, which are often interpreted as reflecting mitochondrial remodeling or compensation in response to altered energetic demands or mitochondrial stress (Castellani et al., 2020; Herbst et al., 2021; Picard et al., 2018). To quantify mtDNA copy number in isolated FDB fibers, we measured mtDNA-encoded genes (16S rRNA, ND1, or a unique mtDNA region-mMito) relative to nuclear genes (HK2 or B2M) by qPCR Across primer sets, the comparison of the fold-change (aged relative to young) in mtDNA content consistently increased with age in both sexes. In addition, aged males exhibited higher mtDNA content than aged females (Figures 1B–D).

### 3.2 Cellular respiratory capacity in FDB muscle undergoes greater changes with age in males relative to females

**FIGURE 2:**
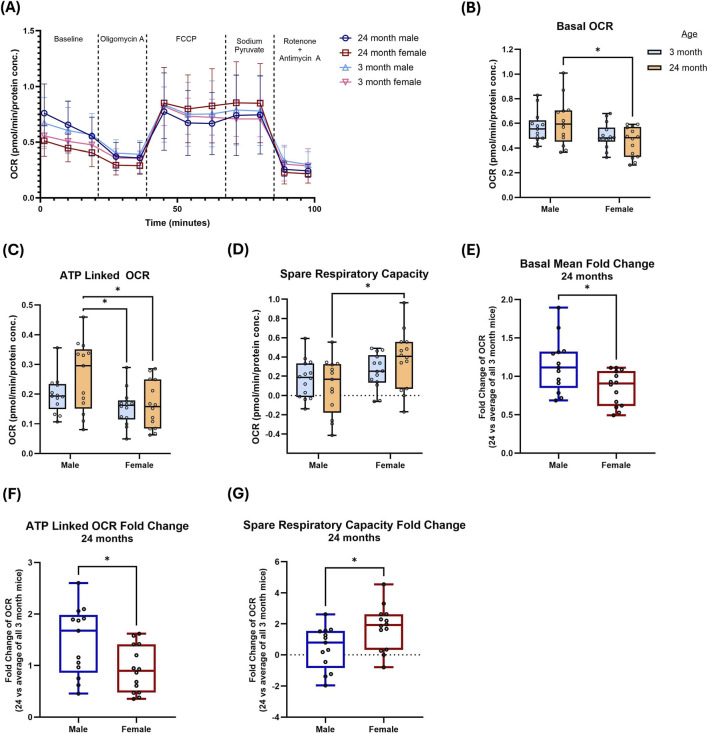
Alterations in cellular oxygen consumption rate between young and aged males and females **(A)** Oxygen consumption rate (OCR) trace in isolated FDB fibers from young (3-month) and aged (24-month) male and female mice. OCR is shown normalized to protein concentration. **(B–D)** Basal OCR, ATP-linked OCR, and spare respiratory capacity. **(E–G)** Fold-change for basal OCR, ATP-linked OCR, and spare respiratory capacity in aged (24-month) mice relative to the mean of young (3-month) mice. Box plots show median (center line) and interquartile range (box); whiskers show [min–max]. Points represent biological replicates (individual mice, n = 12–14 per group). Statistics: two-way ANOVA and one-way ANOVA with Tukey’s multiple comparisons test. *P < 0.05.

Given our observed differences in mtDNA content with age in both sexes as well as the greater age-related changes in males, we next used the Seahorse mitochondrial stress test to quantify mitochondrial respiratory rates (Figure 2A). Basal OCR was lower in females, relative to males, for both age classes though there was a greater difference between sexes in aged individuals (Figure 2B). ATP-linked respiration was lower in aged females relative to aged males (Figure 2C). Maximal OCR, as well as sodium pyruvate–stimulated OCR (a secondary readout of maximal respiratory capacity), showed no significant differences by age or sex (Supplementary Figure S1A,B,D,E). Notably, spare respiratory capacity was increased with age in females and was significantly higher in aged females than aged males (Figure 2D), whereas proton leak OCR was not significantly altered (Supplementary Figure S1C,F).

To visualize age-associated remodeling within each sex, we calculated fold change (aged relative to young) for each OCR parameter. Basal and ATP-linked OCR exhibited sex differences, with aged males showing greater positive fold change compared to the females, which were relatively stable with age (Figures 2E,F). In contrast, the fold change in spare respiratory capacity was significantly higher and positive in females relative to males (Figure 2G). Together, these results suggest distinct changes in the bioenergetic reserve in males and females during aging.

### 3.3 Age- and sex-dependent changes in mitochondrial dynamics, ETC, and mtDNA maintenance gene expression

**FIGURE 3:**
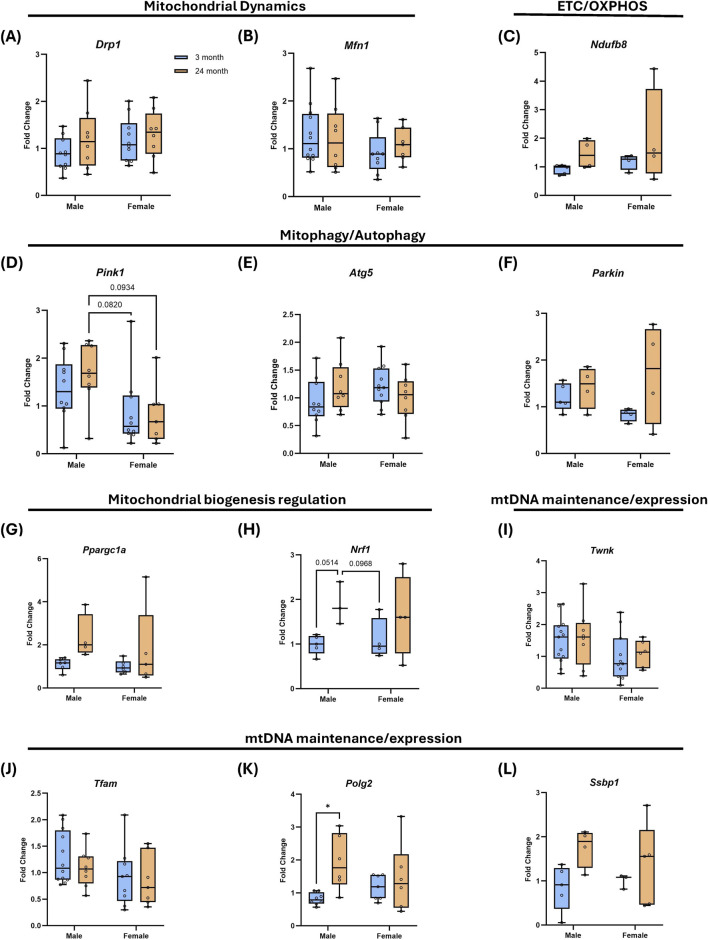
Age- and sex-dependent analysis of gene expression associated with mitochondrial dynamics and function in mouse skeletal muscle. Gene expression in FDB was assessed by RT-qPCR for pathways related to mitochondrial dynamics, electron transport chain function, mitophagy/autophagy, mitochondrial biogenesis regulation and mtDNA maintenance. **(A–L)** Relative expression (fold change) for *Drp1, Mfn1, Ndufb8, Pink1, Atg5, Parkin, Ppargc1a, Nrf1, Twnk, Tfam, Polg2 and Ssbp1* calculated by the ΔΔCt method using *beta Actin* and expressed relative to mean of young mice. Box plots show median and interquartile range with individual biological replicates (mice) overlaid. Statistics: two-way ANOVA with Tukey’s multiple comparisons test. *P < 0.05.

To probe molecular pathways that may underlie bioenergetic remodeling, we measured mRNA levels of 15 genes involved in mitochondrial fission/fusion, mitophagy, ETC function, and mtDNA transcription/replication by RT-qPCR in FDB fibers (Figure 3; Supplementary Figure S2).

Overall, expression of many of the genes we tested did not change significantly with age or exhibit sex differences (Figure 3; Supplementary Figure S2). For example, among the genes involved in mitochondrial fission, fusion, and turnover, the fusion gene *Mfn1* showed minimal age-dependent change (Figure 3B), whereas the fission regulator *Drp1/Dnm1l* trended up with age in both sexes (Figure 3A). Mitophagy-associated transcript *Atg5* was largely stable, while *Pink1*, which has been shown to be elevated in male rodent muscle, also trended lower in females compared to males (Figures 3D,E) (Springer-Sapp et al., 2025). Expression of the proteasome-associated transcript *Parkin* has been shown to increase with age in rodent muscle, also trended up with age in both sexes (Figure 3F) (Springer-Sapp et al., 2025).

Among, ETC genes, the nuclear-encoded complex I subunit *Ndufb8* expression trended upward with age (Figure 3C). Similarly, the mtDNA-encoded complex I gene *ND1* also trended toward age-associated induction (Supplementary Figure S2B). *CO1* (complex IV) showed similar age effects (Supplementary Figure S2C).

Finally, several genes involved in mtDNA maintenance displayed sex- and age-dependent patterns. *Tfam* varied modestly but differed slightly between aged males and females (Figure 3J). *Polg2* increased with age in males (Figure 3K), with similar male-specific age-associated reductions for *Tfb2m* and *Ssbp1* trended upward with age in males and females (Figure 3L; Supplementary Figure S2A). *Twnk* exhibited minimal change (Figure 3I). Biogenesis regulator *Ppargc1a* showed modest age-associated increase in males while *Nrf1* showed modest age-associated increases in both males and females.

To assess the overall effect of age independent of sex, we next collapsed fold-change values across males and females. In this combined analysis, genes in the mitochondria dynamics module and nuclear-encoded ETC gene remained largely unchanged with age (Supplementary Figure S3A–C). In contrast, mtDNA-encoded *ND1*, the mitophagy-associated gene *Parkin*, and both biogenesis regulators (*Ppargc1a* and *Nrf1*) showed age-associated increases (Supplementary Figure S3D,H–J). Within the mtDNA maintenance group, only *Polg2* and *Ssbp1* increased with age (Supplementary Figure S3N–O). Together, these results indicated that while most transcripts were stable across sex and age, the dominant signature among genes that changed was modest age-associated upregulation, consistent with the changes we have seen in mitochondrial content and cellular respiration.

### 3.4 Response to DNA damage is altered with age and sex in blood

To assess DNA integrity and responsiveness to oxidative stress, we used the alkaline single cell gel electrophoresis assay (“comet” assay). Due to the multinucleated nature of FDB fibers, mechanical dissociation and nuclei isolation necessary for the comet assay introduces variability and confounds clean comet measurements in this tissue. Therefore, we focused on nucleated blood cells as a technically tractable sample type for detecting baseline and induced damage to DNA in leukocytes under standard assay conditions. Because mature FDB myofibers are multinucleated, comet analysis would require enzymatic dissociation and/or myonuclear isolation, steps that can introduce mechanical DNA damage and increase technical variability, complicating interpretation of baseline and induced damage in this tissue. Accordingly, we performed comet assays on whole-blood leukocytes as a practical surrogate tissue to quantify DNA damage at baseline and after oxidative challenge. This component of the study was intended as a practical surrogate measure of systemic DNA damage responsiveness rather than a direct, mechanistically linked measurement of DNA damage within FDB myonuclei.

**FIGURE 4:**
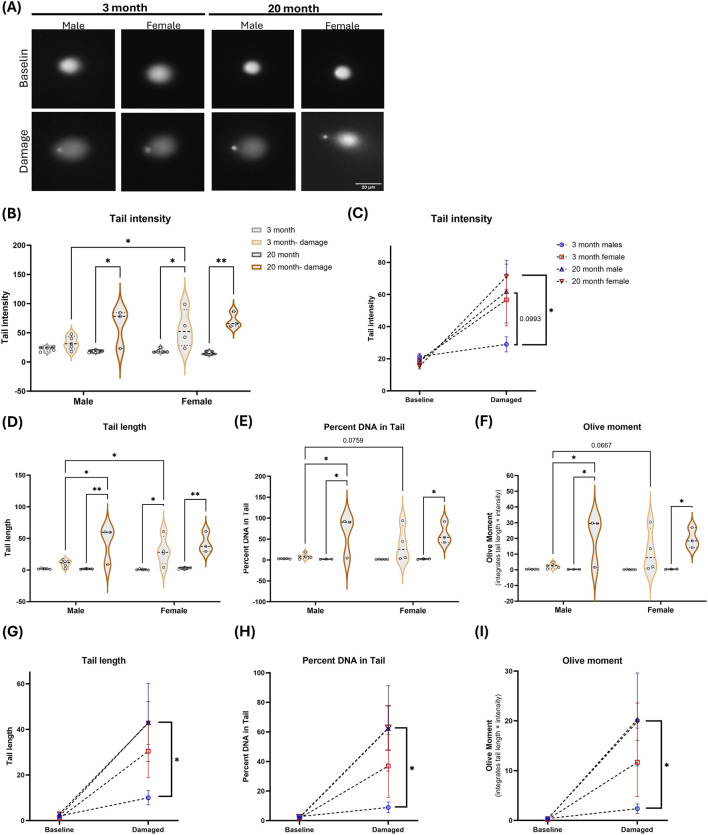
Response to DNA damage is altered with age and sex in the blood. Alkaline comet assays were performed on blood-derived nucleated cells from young (3-month) and aged (20-month) male and female mice under baseline conditions and following DNA damage induction with H_2_O_2_. **(A)** Representative comet images for each sex/age group at baseline and after damage induction. **(B–C)** Tail intensity at baseline and after damage induction shown as group distributions **(B)** and paired/connected group means **(C)**. **(D,G)** Tail length shown as group distributions **(D)** and paired/connected group means **(G)**. **(E,H)** Percent DNA in tail shown as group distributions **(E)** and paired/connected group means **(H)**. **(F,I)** Olive tail moment (integrates tail length × tail intensity) shown as group distributions **(F)** and paired/connected group means **(I)**. Violin plots show distributions with individual data points overlaid representing per-mouse averages n = 5 male/5 female mice for 3 months baseline group; 5 male/4female mice for 3 months damage group; 3 male/3 female mice for 20 months baseline group; 3 male/3 female mice for 20 months damage group (with at least 12 comets per mouse for baseline and at least 4 comets per mouse with damage treatment); connected line plots depict within-group change from baseline to damaged. Statistics: two-way ANOVA with Tukey’s multiple comparisons test. *P < 0.05; **P < 0.01.

In baseline blood samples, comet tail intensity, tail length, percent DNA in tail and olive moment did not differ significantly by age or sex (Figures 4A,B,D–F). Following *ex vivo* oxidative challenge with hydrogen peroxide, tail metrics increased relative to baseline, and young females exhibited higher inducible damage to oxidative stress than young males under the same conditions (Figures 4C,G–I). We did not observe a sex difference in the response to damage in the aged mice. These findings suggest that nucleated blood cells from young male mice are more resilient to oxidative damage relative to young females.

## 4 Discussion

In this study, we provide a multi-parameter assessment of sex- and age-related differences in mitochondrial function and DNA integrity in mouse skeletal muscle, focusing on the FDB and circulating leukocytes. We observed a significant increase in mtDNA copy number in both males and females with age, with a greater increase in males relative to females. Changes in mtDNA content are commonly interpreted as markers of mitochondrial remodeling or compensation and do not by themselves establish dysfunction; in the context of our cellular respiration data, the greater mtDNA expansion in males is consistent with a more pronounced age-associated remodeling of mitochondrial programs in male FDB (Castellani et al., 2020; Herbst et al., 2021; Picard et al., 2018).

Similarly, males exhibited larger age-associated shifts in basal and ATP-linked respiration, alongside modest changes in transcripts related to mitochondrial quality control, biogenesis, and mtDNA maintenance. Notably, gene expression differences across sex and age were generally small, which may reflect limited sample size, inter-individual variability, hormonal cycling may also contribute to within-group variability, and/or regulation at post-transcriptional or functional levels rather than broad transcriptional reprogramming.

The increased spare respiratory capacity in aged females compared to aged males, despite lower baseline OCR, may suggest a greater capacity for female FDB muscle to handle energy-demanding or stressful conditions in the short term. One interpretation is that aged female fibers maintain a larger bioenergetic reserve, potentially reflecting greater substrate flexibility and/or differences in fiber-type composition. If respiratory capacity is preserved, females may require fewer compensatory changes in basal respiration or mtDNA copy number with age compared to males. Consistent with this, females exhibited fewer age-associated changes to basal respiration or mtDNA content.

As a secondary analysis, we assessed baseline DNA integrity and responses to oxidative challenge in whole-blood leukocytes using the alkaline comet assay. Young females displayed greater oxidative damage metrics than young males after oxidative stress challenge in blood. Because mature FDB myofibers are multinucleated, comet analysis would require enzymatic dissociation and/or myonuclear isolation, which can introduce mechanical DNA damage and increase technical variability. We therefore used whole-blood nucleated cells (leukocytes) as a practical surrogate tissue to quantify baseline and inducible DNA damage under standardized assay conditions; accordingly, these data should be interpreted as a systemic leukocyte readout rather than a direct measure of DNA integrity in FDB myonuclei. Whether this reflects increased susceptibility to oxidative damage, differences in repair kinetics, or cell-type composition differences in blood will require additional targeted follow-up studies. In skeletal muscle, complementary approaches for quantifying nuclear and mtDNA lesions in fibers, such as optimized nuclei isolation, long-amplicon qPCR, or immunostaining for DNA damage response proteins may be better suited for directly assessing nuclear and mitochondrial genome integrity in fibers.

Collectively, our findings underscore the importance of considering sex as a biological variable when interrogating muscle bioenergetics across the lifespan and motivate future work to define the mechanisms driving sex-specific aging trajectories and their relationship to systemic DNA damage responses.

## Data Availability

Publicly available datasets were analyzed in this study. This data can be found here: Holcom, Angelina (2026), “Sex Differences in Mitochondrial Function in Aging Mouse Skeletal Muscle”, Mendeley Data, V2, doi: 10.17632/8hz9kxyj2c.2.

## Supplementary material

SUPPLEMENTARY FIGURE S1 Mitochondrial maximum and proton leak oxygen consumption rate were not altered with age or sex. **(a–c)** Quantification of maximum OCR, sodium pyruvate–induced OCR, and proton leak OCR from Seahorse measurements in isolated FDB fibers (3-month vs. 24-month; male vs. female). **(d–f)** Fold-change analyses for maximum OCR, sodium pyruvate–induced OCR, and proton leak OCR in aged (24-month) mice relative to the mean of young (3-month) mice. Box plots show median and interquartile range with individual biological replicates overlaid. Statistics: two-way ANOVA and one-ANOVA with Tukey’s multiple comparisons test.

SUPPLEMENTARY FIGURE S2 Age-related changes in genes associated with mtDNA transcription ETC complex 1 and 4. RT-qPCR was performed for genes related to mtDNA transcription initiation (*Tf2bm*) and mitochondrial-encoded ETC genes *ND1* and *CO1*. **(a–c)** Relative expression (fold change) calculated by ΔΔCt using *beta Actin* and expressed relative to mean of young mice. Box plots show median and interquartile range with individual mice overlaid. Statistics: two-way ANOVA with Tukey’s multiple comparisons test.

SUPPLEMENTARY FIGURE S3 Age-dependent fold changes in mitochondrial pathway genes in FDB (males and females pooled). To assess the main effect of age independent of sex and increase power to detect age-associated changes, RT–qPCR fold-change values from Figure 3 and Supplementary Figure S2 were collapsed across males and females. **(a–o)** Relative expression (fold change) was calculated by ΔΔCt using *beta Actin* expressed relative to mean of young mice. For the sex-collapsed analysis, per-mouse fold-change values were combined across males and females within each age group, yielding two comparison groups (young vs. aged).
