# Supplementary figures and images for: Sex differences in mitochondrial function in aging mouse skeletal muscle
Source: Front Aging. 2026 Apr 29;7:1824237. doi: 10.3389/fragi.2026.1824237 (PMC13167975; doi:10.3389/fragi.2026.1824237)

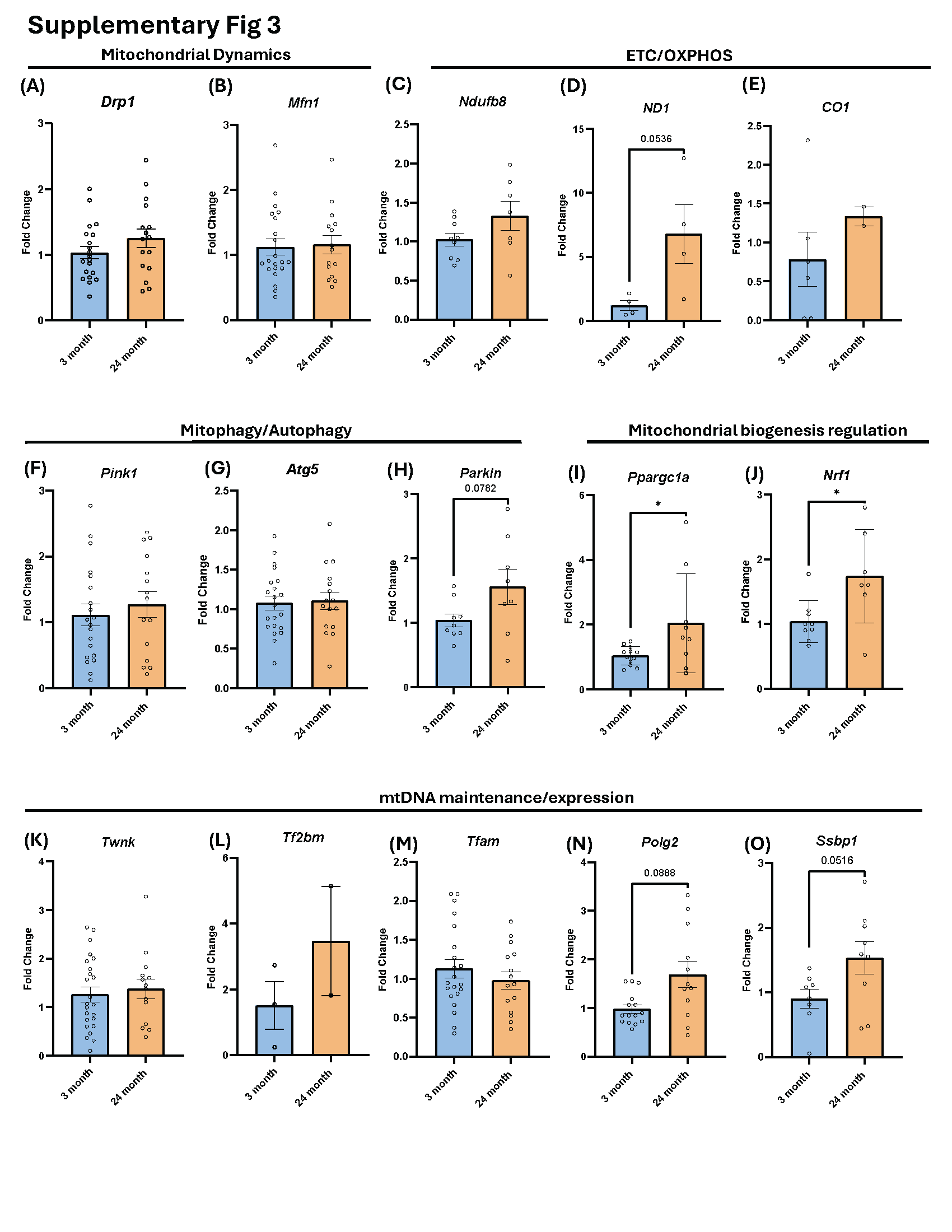

Supplement: Supplementary file 1 [file Image3.tiff]

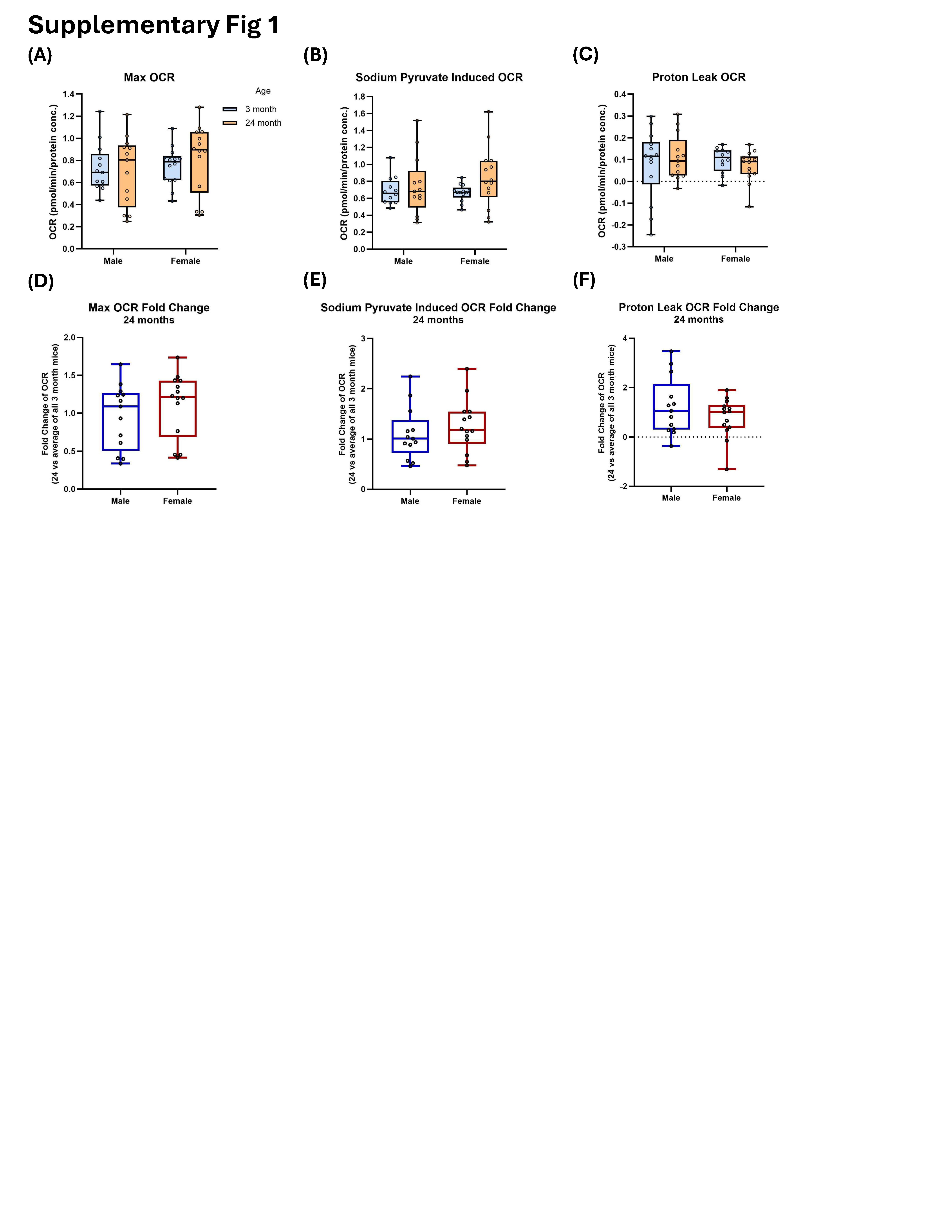

Supplement: Supplementary file 2 [file Image1.tiff]

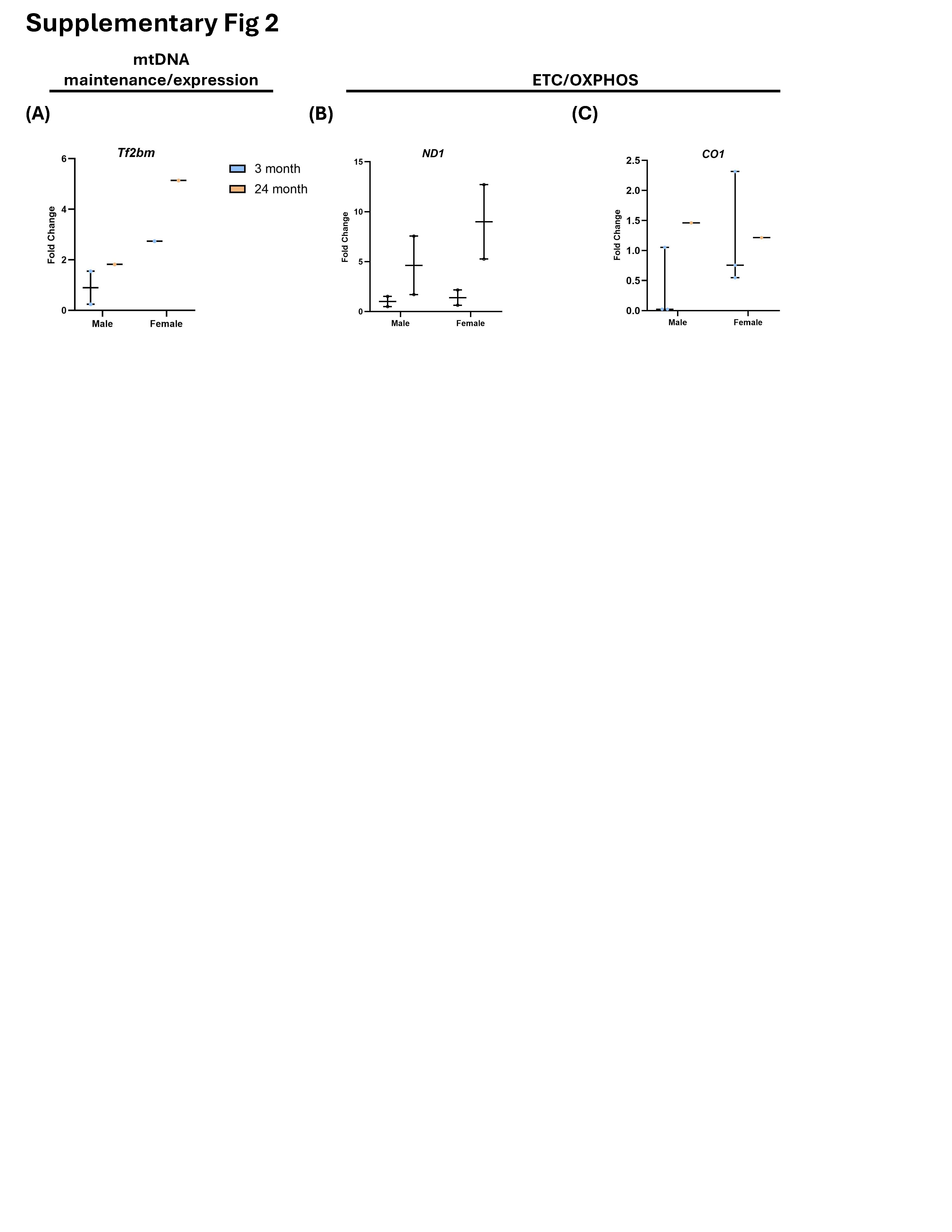

Supplement: Supplementary file 3 [file Image2.tiff]
